# Machine Learning-Devised Immune-Related lncRNA Signature Panel Predicts the Prognosis and Immune Landscape in Breast Cancer Novel IRLP Signature in BRCA

**DOI:** 10.1155/2022/3704798

**Published:** 2022-08-18

**Authors:** Jun-Yu Zhu, An-Qi Lyu, Zhang-Ting Wang, Wai-Yee Chan, Tao Qin, Kai-Kei Miu, He-Rui Yao

**Affiliations:** ^1^Centre of Phase I Clinical Trial, Department of Medical Oncology, Sun Yat-Sen Memorial Hospital, Sun Yat-Sen University, Guangzhou, Guangdong 510000, China; ^2^School of Biomedical Sciences, Faculty of Medicine, The Chinese University of Hong Kong, Hong Kong SAR 999077, China

## Abstract

Long noncoding RNAs (lncRNAs) actively participate in breast cancer (BRCA) tumorigenesis via epigenetic mechanisms. Our study identified immune-related lncRNA (irlncRNA) pairs and compiled them into a set of noncoding gene signatures able to stratify subtypes of BRCA associated with variable degrees of survival and immune cell infiltration. A 40 immune-related lncRNA pair (IRLP) signature including 43 irlncRNAs was built, with high sensitivity and specificity for the prediction of survival in different molecular subtypes of BRCA. Results demonstrated that the low-risk group showed a significantly longer survival rate, and this novel IRLP signature was highly associated with survival status, T stage, metastatic disease, and overall stage in BRCA. Immune infiltrating analyses found that the low-risk group has a lower expression level of macrophage M2 and a higher expression level of immunosuppressed biomarkers than the high-risk group. DEirlncRNAs were further proven to be significantly related to the MAPK signaling, Jak-STAT signaling, and ErbB signaling pathways in BRCA. In conclusion, the 40 IRLP signature showed a promising clinical prediction value in the prognosis of different molecular subtypes and immunotherapy response in BRCA, and the underlying mechanism for these IRLPs warrants further investigations.

## 1. Introduction

Breast cancer ranks first for global cancer incidence in 110 countries in 2020 [1]. Despite the high incidence, breast cancer manifests its molecular mechanisms divergently and therefore represents a highly heterogeneous type of cancer. With an overt lack of molecular signatures that may effectively stratify tumor subclasses, there are also devoid gene panels to predict personalized therapeutic effects.

Standard treatment of breast cancer relied heavily on a combination use of chemotherapeutics and hormonal disrupting agents in accordance with either their hormonal receptor (PR, ER) expression or their pathway-related molecular subtypes. In recent years, advanced translational research resulted in immune checkpoint inhibitors (ICIs) and modulators with some being investigated in breast cancer management. The contemporary guidance for drug usage depends on molecular diagnosis in monitoring gene signatures comprised of coding gene expression, mutational status, and DNA damage-related epigenetic phenomena. For example, high microsatellite instability (MSI-H), programmed death-1 (PD-1), programmed cell death ligand 1 (PD-L1), tumor mutational burden, and mismatch repair deficient (dMMR) have been widely used in clinic as predictive biomarkers for ICIs. With that, one may project the importance of gene modulators that modifies the immune cell niche as prognostic markers for BRCA.

The immunotherapy field had achieved an immense breakthrough in the treatment of several cancer types [2]. Even though a defined set of predictive markers was adopted in those cancers, the same set tends to resist corresponding changes in breast cancer, limiting its predictive value. A lack of personalized treatment indication had been reflected in immunotherapy receiving only a modest response rate in breast cancer [3]. Therefore, there is an urge to optimize this meager outcome-based prediction method to enhance the clinical response rate of breast cancer immunotherapy.

lncRNAs are active participants in breast cancer tumorigenesis via epigenetic mechanisms, rendering them valuable predictive markers in this cancer progression and treatment. As a loose term for the entire collection of intracellular noncoding RNAs that were over 200 nucleotides, lncRNA demonstrates a myriad of actions implicated in regulating over 70% of coding gene expression through direct interactions or sponge effects [4]. A subclass of lncRNAs directly participated in the remodeling of the tumor immune microenvironment (TIME), resulting in augmented tumor malignancy and tumor immune escape [5]. The lncRNA GNAS-AS1 also sets an exemplary example for the conserved actions of its class in modulating immune surveillance of breast cancer. GNAS-AS1 can motivate M2 macrophage polarization in situ via GATA3 activation in the estrogen receptor-positive (ER-positive) tumors [6]. Similar M2 polarization was also reported along with heightened noncoding BCRT1 expression [7]. In addition, other modes of immunomodulatory actions by these lncRNAs were reported to be dysregulated in the cancerous lesion. For example, a lncRNA named NKILA directly sensitized T cells to induce the progression of activation-induced cell death (AICD) and lead to tumor immune evasion [8]. Collectively, these immune-related lncRNAs (irlncRNAs) can be further enriched and weighed to construct a signature panel for a better prognosis and prediction of clinical response of ICIs [9–11].

In our study, we intend to explore the predictive power of this overlooked collection of noncoding factors at the transcript level, particularly over how they may exert actions over immune surveillance, to reflect the likelihood of treatment responses and anticipate probable refractory cases. We sought to build an irlncRNAs-related computational model which efficiently predicts the immune landscape of breast cancer prognosis in different molecular subtypes. Our project also extended to investigate the functions of those with differential expression profiles across the subtypes.

## 2. Materials and Methods

### 2.1. Transcriptome Data Collection and DEirlncRNA Verification

We downloaded the fragments per kilobase per million (FPKM) of normal tissue and breast cancer transcriptome profiling and metadata of diagnosed patients in these categories from The Cancer Genome Atlas (TCGA) database in May 2021 (Table S1). Subsequently, immune-related genes were downloaded from the ImmPort database. GTF file (genecode v23) of hg19 was used to distinguish lncRNAs and mRNAs in our study.

### 2.2. Processing of RNA-seq Data

Coexpression analyses were performed to identify the irlncRNAs. The lncRNAs that met the cut-off criteria of correlation coefficients > 0.4 and *p* value < 0.001 with ir-mRNAs were distinguished as irlncRNAs, and the irlncRNAs with ∣log FC | >1.5 and FDR < 0.5 were identified as DEirlncRNAs. A 0 or 1 matrix was formed 0 as the expression of the former lncRNA in the DEirlncRNA pair of which its expression is lower than the latter, while 1 is indicative of the relative higher expression in the former. Qualified DEirlncRNA pairs shall hold a skewed proportion to having either 0 or 1 as higher than 20% in the matrix.

### 2.3. Development of a Risk Score (RS)

The valid DEirlncRNA pairs were screened using uni-Cox analysis; the threshold is *p* < 0.05. Then, R package glmnet was performed for building the Lasso model with 10-fold cross-validation [12]. A multi-Cox analysis was used to infer the definitive parameters of each prognostic factor. The risk score = ∑_*i*=1_^*k*^*βiSi*.

### 2.4. RS Validation

Our study also calculated the AUC values of each model, and R package ROCR was used for generating the receiver operating characteristic (ROC) curve plots of 1, 3, and 5 years [13]. Then, we validate the cut-off point by Kaplan-Meier analysis.

### 2.5. Comparison between RS and Clinical Features

To confirm the clinical value of our novel model, the paired *t*-test is performed between the signature and clinicopathological features. Survival status, age, gender, metastatic disease, N stage, T stage, and clinical stage were considered variables. To further verify if the RS can serve as an individual predictor, uni-Cox and multi-Cox were conducted between the high- and low-RS groups.

### 2.6. Patient Subtyping and Immune Infiltration

BRCA is usually divided into 4 molecular subtypes—Her2-enriched, luminal A, luminal B, and triple-negative breast cancer (TNBC). For patients without the metadata of intrinsic molecular subtypes, PAM50 function was used for classifying the molecular subtypes [14]. The survival plot of the low-risk and high-risk groups in PAM50 subtypes was also plotted. Other currently acknowledged functions (ssp2006, scmgene) were also applied to classify the subtypes. The differences between high-risk and low-risk group numbers in those subtypes were analysed by a chi-squared test. To reveal the distinct immune infiltration patterns of different subtypes, the immune infiltration statues between the high-risk and low-risk groups of constructed models were analysed by CIBERSORTX analysis [15].

### 2.7. Enrichment Analysis

To infer the possible biological functional pathway of irlncRNAs in the prognostic signature, RNA central [16] was used to perform the GO analysis. The GO of protein-coding genes correlated with the irlncRNAs were also predicted using MSigDb v7.2 Hallmarks [17] and R package clusterProfiler v3.10.1 [18].

### 2.8. Statistical Analysis

The R package ggplot2 and ggpubr package was performed to produce the plots. A *p* value < 0.05 was considered significant in our study, where visualization was labelled as below: ^∗∗∗^<0.001, ^∗∗∗^<0.01, and ^∗^<0.05.

## 3. Results

### 3.1. DEirlncRNA Identification

**Figure 1:**
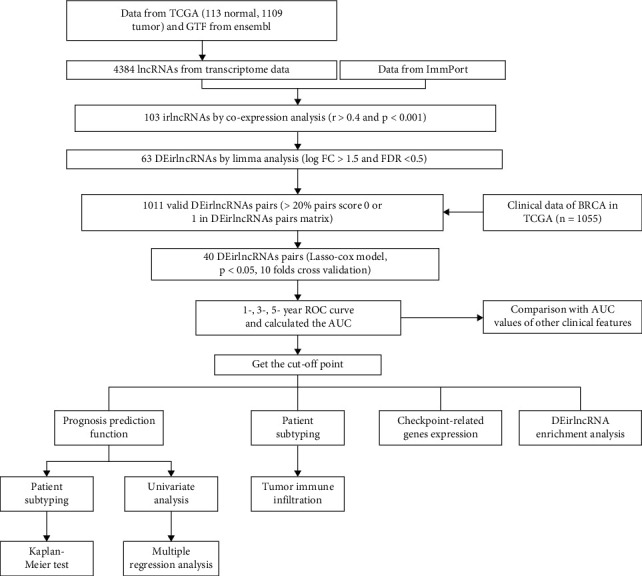
Study flowchart for constructing the signature.

**Figure 2:**
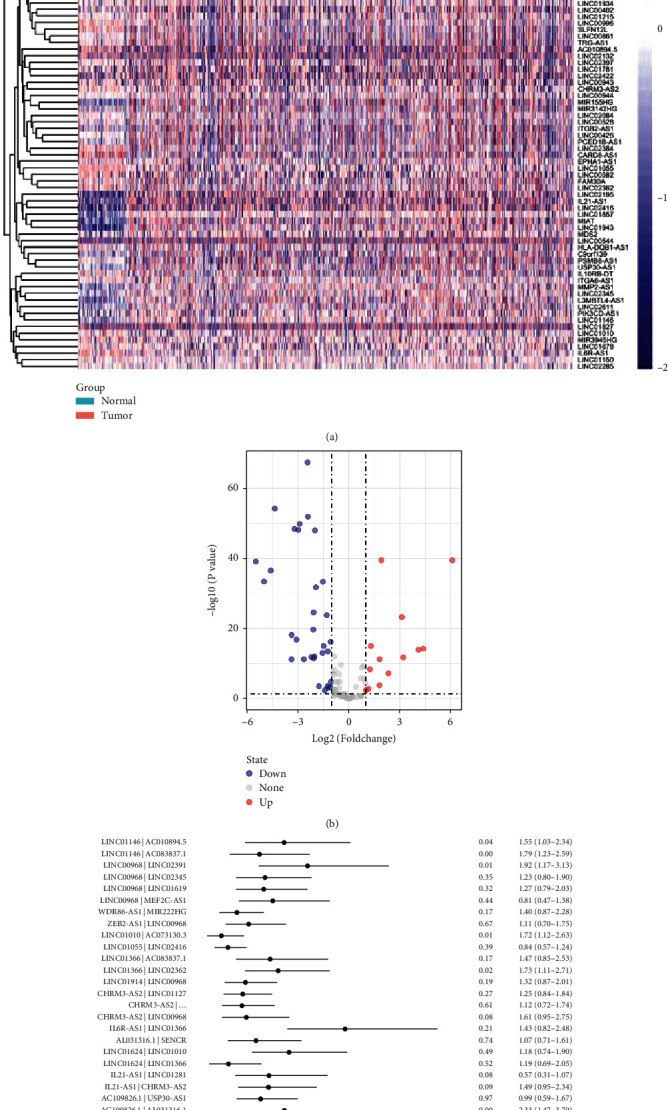
Establishment of a risk assessment model. The heat map of 103 irlncRNAs (a) and a volcano plot of 63 DEirlncRNAs (b) are shown. The forest map showed the details of the 40 IRLPs (c).

Firstly, we obtained the transcriptomic profiles of 113 normal and 1109 tumor cases from TCGA database. Those data were annotated with gene transfer format (GTF) files downloaded from the Ensembl database (Figure 1). In further coexpression analysis between lncRNAs and immune-related genes, we have screened out 103 irlncRNAs (Figure 2(a)) and 63 DEirlncRNAs, among which 45 were downregulated and 18 were upregulated in BRCA (Figure 2(b)).

### 3.2. Screening DEirlncRNA Pairs and Establishing Risk Assessment Signature

**Figure 3:**
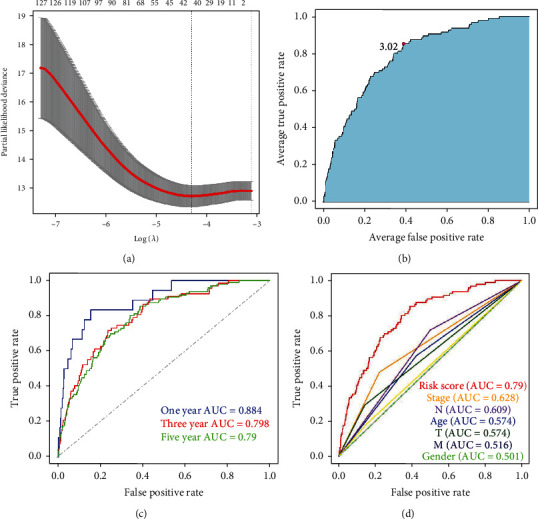
Diagrams of Lasso regression analysis and multi-Cox regression analysis. Lasso regression analysis (a). Identifying the highest point of the AUC by ROCs of 40 IRLP model (b). 1-, 3-, and 5-year ROC showing all AUC values were around 0.80 (c). A comparison of 5-year ROC curves with other BRCA survival-related clinicopathological features showed the superiority of the RS (d).

63 DEirlncRNAs produced 1011 valid DEirlncRNA pairs. Then, 40 DEirlncRNA pairs were screened by Lasso regression analysis from 1011 valid DEirlncRNA pairs (Figure 2(c)) for signature establishment. Then, we subjected those 40 DEirlncRNA pairs to multi-Cox regression analysis (stepwise method) for confirming such signature establishment (Figure 3(a)). The identified cut-off point of 5-year ROC curves was applied to separate individuals into the high-risk and low-risk groups (Figure 3(b)). Afterward, the predictive capacity of this risk assessment signature was applied for the 1-, 3-, and 5-year ROC curves. The AUCs of those ROC curves were over 0.79 indicating that this novel signature works well in cancer survival rate prediction (Figure 3(c)). Furthermore, we detected the ROC curves of 5-year and other reported clinicopathological features. The AUCs demonstrated that our risk assessment model is an optimal predictor for the prognosis of BRCA (Figure 3(d)).

### 3.3. BRCA Molecular Subtype Identification and Clinical Evaluation by IRLP Signature

**Figure 4:**
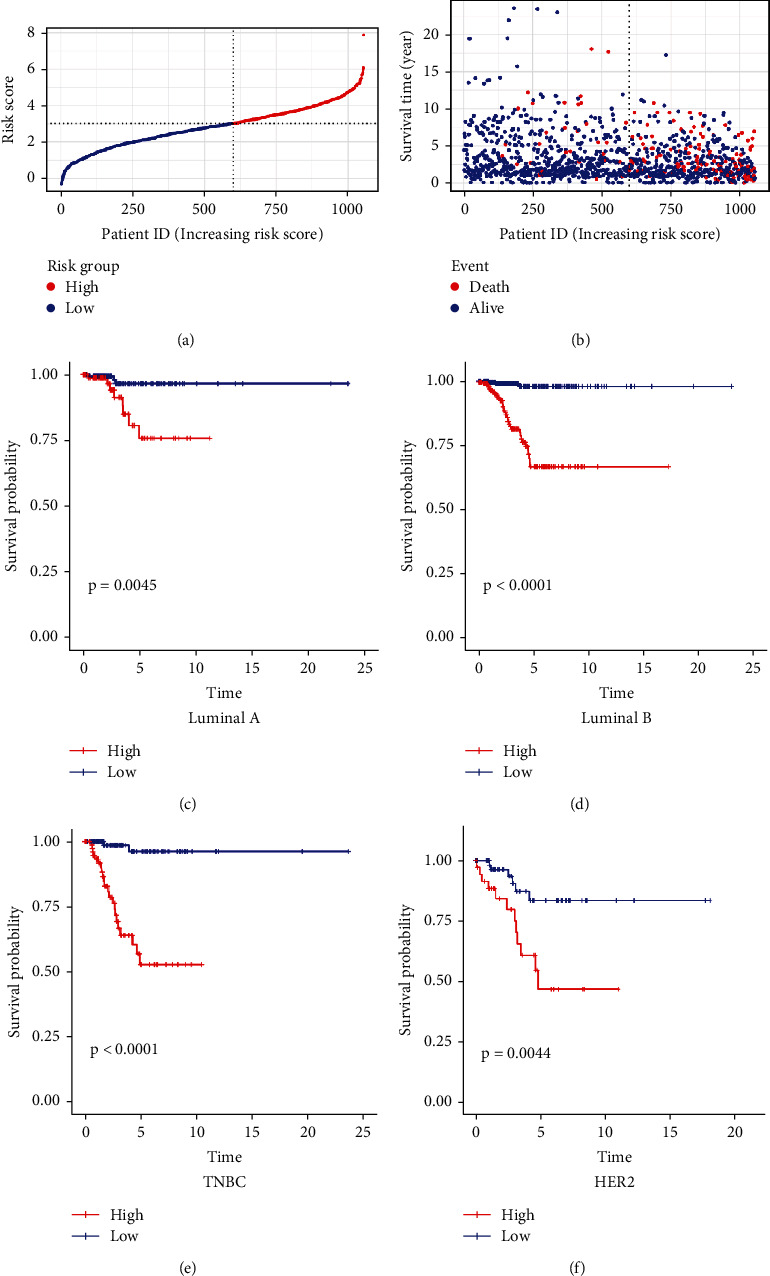
IRLP signature for prognosis prediction of the patient with BRCA. RS (a) and survival outcome (b) of patients with breast cancer are shown. Patients in the low-risk group experienced a longer survival time in all subtypes.

1055 eligible cases were enrolled from the 1109 tumor cases for survival analysis. There are a total of 455 cases distinguished into the low-risk group and 600 in the high-risk group. RS and the survival status are calculated (Figures 4(a) and 4(b)). Since the survival time of BRCA is verified in different molecular subtypes, Kaplan-Meier analysis was investigated in four molecular subtypes. A total of 197, 106, 504, 228, and 20 patients were identified as TNBC, Her2, luminal A, and luminal B in PAM50 to corresponding subtypes (low RSG: 106, 69, 258, 142, and 13, respectively; high RSG: 86, 37, 241, 83, and 7, respectively). In addition, patients in the low-risk group exhibited a longer survival time in all molecular subtypes (*p* < 0.001) (Figures 4(c)–4(f)), indicating that our signature is suitable for all patients with breast cancer.

**Figure 5:**
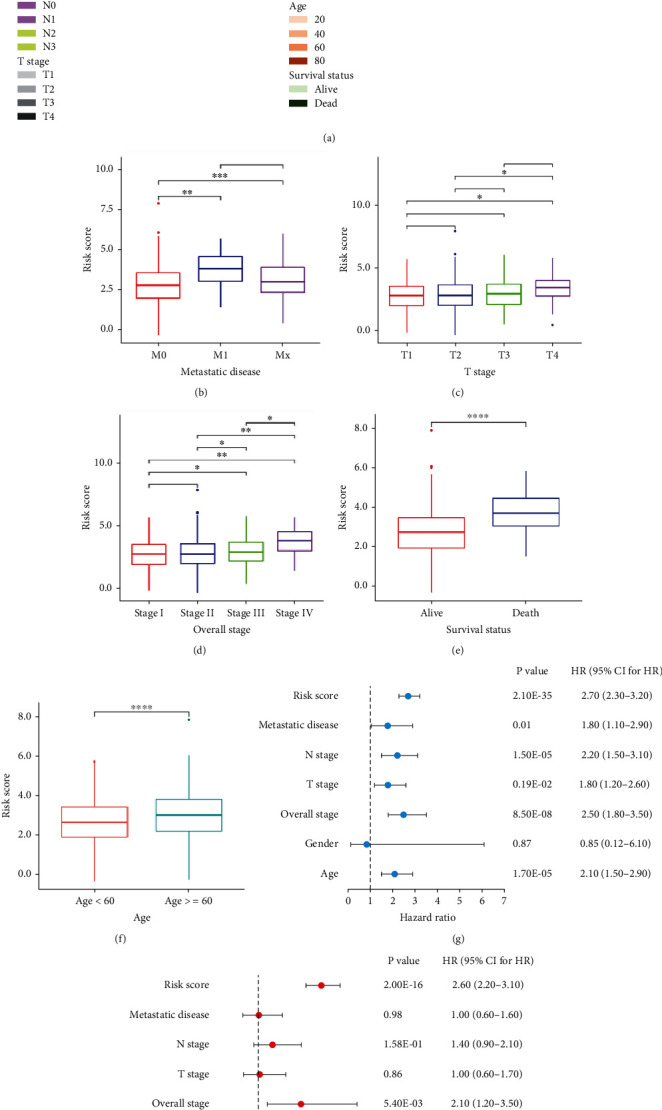
Application of the IRLP signature for clinical evaluation. Strip chart showing the clinical information of each case (a). Metastatic disease stage (b), T stage (c), clinical stage (d), survival status (e), and age (f) were significantly associated with the RS. A uni-Cox hazard ratio analysis demonstrated that risk score (*p* < 0.001, HR = 2.70, 95%CI [2.30–3.20]), N stage (*p* < 0.001, HR = 2.20, 95%CI [1.50–3.10]), T stage (*p* < 0.05, HR = 1.80, 95%CI [1.20–2.60]), overall stage (*p* < 0.001, HR = 2.50, 95%CI [1.80–3.50]), and age (*p* < 0.001, HR = 2.10, 95%CI [1.50–2.90]) (g). Multi-Cox regression showing that risk score (*p* < 0.001, HR = 2.60, 95%CI [2.20–3.10]), overall stage (*p* < 0.05, HR = 2.10, 95%CI [1.20–3.50]), and age (*p* < 0.05, HR = 1.50, 95%CI [1.10–2.20]) (h).

The further nonparametric Wilcoxon signed-rank test also demonstrated that metastatic disease, T stage, clinical stage, survival status, and age were significantly associated with the heightened cancer risk (Figure 5). Therefore, we investigated the statistical differences between those clinical factors and risk score by uni-Cox (Figure 5(g)) and multi-Cox (Figure 5(h)) regression analyses. Results showed that risk score, overall stage, and age all exhibited statistical differences.

### 3.4. Investigation of Tumor-Infiltrating Immune Cells (TIICs) and Immunosuppression-Related Genes

**Figure 6:**
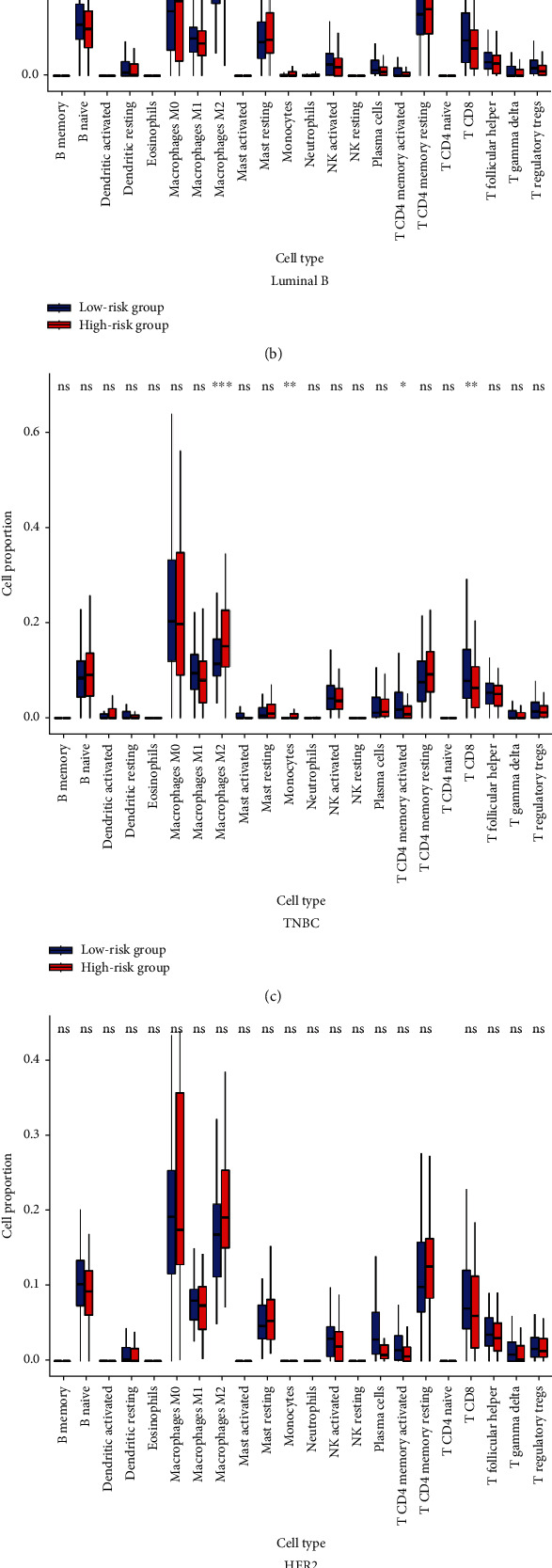
Evaluations of TIICs and immunosuppression-related genes by the IRLP signature. CIBERSORT showed that the high-risk group was positively associated with a higher infiltration level of macrophages M2/M0 and negatively associated with CD8^+^ T cells in all subtypes including luminal A (a), luminal B (b), TNBC (c), and HER2 (d). High-risk group was positively correlated with higher expression levels of LAG3 (e), CTLA4 (f), PDCD1 (g), and PDCD1LG2 (h).

According to the patient subtypes, correlation analysis showed that the high-risk group was positively associated with higher infiltration levels of macrophages M2/M0 and negatively associated with CD8^+^ T cell infiltration in luminal A (Figure 6(a), Table S2A), luminal B (Figure 6(b), Table S2B), and basal (Figure 6(c), Table S2C). However, TIICs showed no significant difference in HER2 BRCA (Figure 6(d), Table S2D). Furthermore, we used Spearman correlation analysis to investigate whether the IRLP signature was related to immunosuppression-related biomarkers in all patients with breast cancer. The analysis revealed that the high-risk group was positively correlated with lower expression levels of LAG3, CTLA4, PDCD1, and PDCD1LG2 (Figure 6).

### 3.5. Identification of 43 irlncRNA Signature Associated with Biological Processes

**Figure 7:**
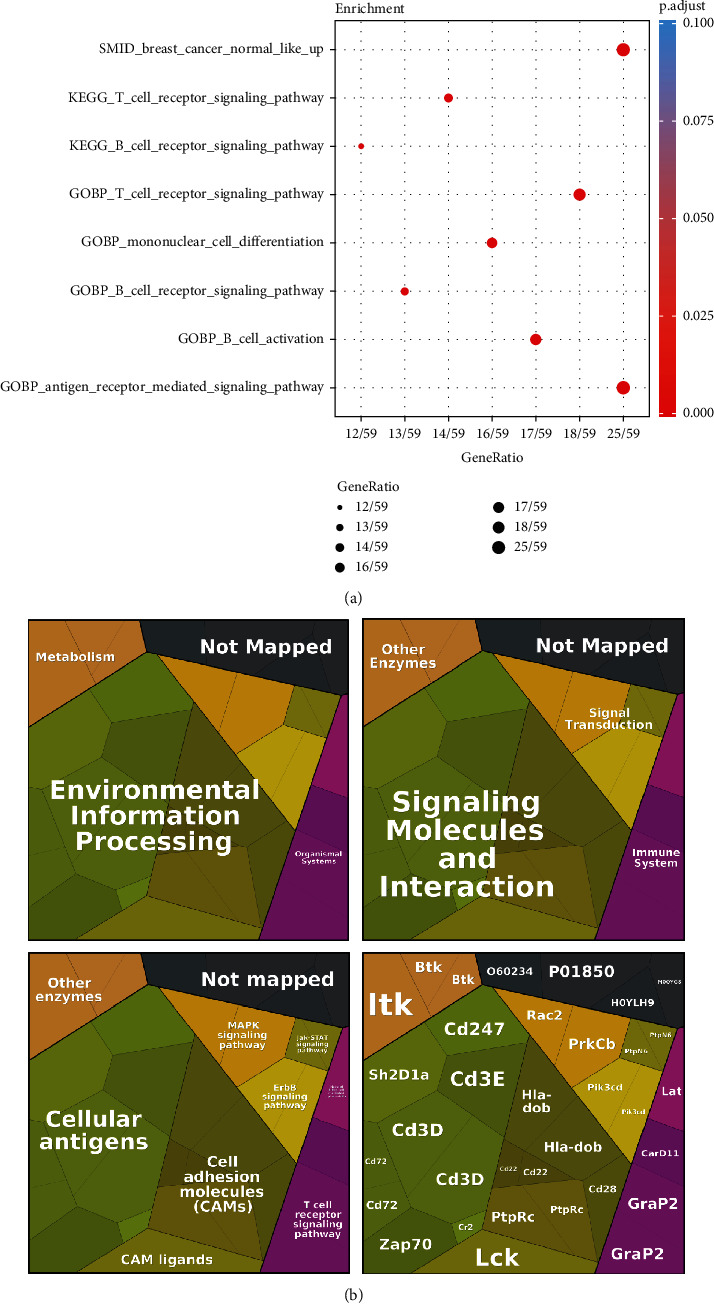
Functional analysis and identification of mutation landscape. GO enrichment between the high-risk and low-risk groups (a). Online Proteomaps pathway analysis of the identified 43 irlncRNAs (b). The third hierarchy level reveals those lncRNAs involved in NQO1 pathways, MAPK signaling pathway, ErbB signaling pathway, and Jak-STAT signaling pathway. The lowest level shows the individual proteins.

Finally, we investigated the mechanisms of these 43 irlncRNAs from the 40 DEirlncRNA pairs signature. The mRNA expression profiles were downloaded from TCGA database and analysed with Pearson correlation to screen out potential genes which are highly correlated with the 43 irlncRNAs (∣*R* | >0.6 and *p* < 0.05). Those identified genes were further subject to GO enrichment analysis (Figure 7(a), Table S3) and Proteomaps pathway analysis to classify the functions of those mRNAs. Results showed that genes associated with the 43 irlncRNAs were clustered into the NQO1, cellular antigens, cell adhesion molecules (CAMs), MAPK signaling, Jak-STAT signaling, ErbB signaling, and Jak-STAT signaling pathways (Figure 7(b)).

## 4. Discussion

Breast cancer is defined as an immunogenic “cold” tumor in clinical settings. As in hepatocellular carcinoma (HCC) and head and neck cancer niches, T cell-derived immune privileges demonstrate worsen prognosis along with immunotherapeutic regimes. The proven hardships in the application of ICIs in breast cancer types had dwindled continuous research interest in immunotherapy. Furthermore, coupled with a lack of an effective immune gene signature for the predictive outcome, the apparent pharmacological idiosyncrasy further hampered the development of personalized regimes as the last resort in breast cancer treatment. Indeed, in ICI therapy as in all other personalized treatment, precision medicine approaches by molecular profiling remains the key to improving all therapeutic success. As a result, it must be emphasized that the medical status quo might have indirectly caused an underaddressed call in the use of ICIs in combating the disease, particularly those highly immune infiltrated subtypes per se.

The evaluation of the expression level of PD-1/PD-L1 and the presence and activation status of tumor-infiltrating T cells are promising tools to improve the effect of tumor immunotherapy [2, 19]. However, since BRCA is a type of immunosuppressive cancer, more sensitive predictive biomarkers are needed for predicting the efficacy of ICIs. Devising alternative means to probe TIICs or immunosuppression-related gene expression remains the most zealous means to predict the ICI responsive rate in BRCA; therefore, we suggest noncoding molecular changes as surrogate immune biomarkers instead. With our current computational model, we reinstated the use of lncRNA gene panel to make a prognosis of breast cancer, in particular for those irlncRNAs regulating tumor immune status in situ.

Nevertheless, lncRNAs were implicated in driving malignant phenotypes in almost all walks of cancer types, and many such transcripts formed the basis for prognostic expression signatures of BRCA with merit success. For example, Shen et al. identified 11 lncRNAs as a prognostic signature for BRCA [20], and Ma et al. established a signature for predicting the survival of BRCA based on the expression levels of 8 irlncRNAs [21]. In addition, a five-irlncRNA signature is also built as a prognostic model for BRCA patients [22]. However, the limitation to those studies was that those revealed lncRNAs were often widely expressed with promiscuous action.

Indeed, there were past attempts to devise expression profiles for constructing irlncRNA prognostic signatures for breast cancer. However, previous signatures are mostly based on quantifying transcript expression levels and seemingly ignored the high difference between heterogeneous biological and clinical features in molecular subtypes. Moreover, in coding genes categorized by the molecular pathway, they partake in; the biological function of those gene modulators could be revealed from how they interact with their binding partners directly. Unlike those coding gene products, most lncRNA driven by its mode of action as antisense transcripts tend to bind multiple target mRNAs while its presence may lead to variant expression changes of the binding partner through both direct and indirect interactions.

A highlight in the current study is an emphasis on filtering out only reasonable signatures based on irlncRNA pairings and discriminating the unified or divergent function of these enriched gene sets across different molecular subtypes. It remains difficult to single out a precise molecular action for any lncRNA merely by its own expression, and therefore, we sought to rely on pinpointed coexpression pairs. Their largely uncharted molecular functions also prompted us to explore the stage-specific actions of our clustered DEirlncRNA pairs along with the immune feature-stratified disease subtypes.

lncRNA pairs are a reasonable optimized method for building a prognosis signature compared to single lncRNAs. lncRNA pairs combined with higher or lower expression lncRNAs ignore their exact expression levels in the signature, which renders detecting specific expression values of all irlncRNA in the expression profile unnecessary. Indeed, such lncRNA pair signature had shown selectively greater advantage than the transcriptome approach in predictive performance in various cancers [9, 10]. Then, Qin et al. build a 33-IRLP signature to predict the immune landscape of breast cancer. However, they did not compare the difference between different molecular subtypes of BRCA [23]. In this study, based on the BRCA DEirlncRNA dataset, a 40 IRLP signature was constructed as an independent prognostic predictor for BRCA. We firstly investigate the effect of this signature on the different molecular subtypes of BRCA, and our results approved this model as a suitable approach to discriminate all 4 major molecular subtypes—luminal A, luminal B, TNBC, and HER2.

Some DEirlncRNAs in the 40 IRLP signature have been validated to be involved in the occurrence and development of tumors. lncZEB2-AS1 and MEF2C-AS1 have been demonstrated as poor biomarkers for patients with BRCA [24, 25]. Our data indicated those IRLPs are in particular related to MAPK signaling, Jak-STAT signaling, and ErbB signaling pathways in BRCA. All these pathways have been found to participate in the progression of proliferation, cancer apoptosis, and cell cycle. The ErbB family members are important for the initiation and maintenance of certain solid tumors, by targeting the HER2-HER3 oncogenic factors via the PI3K/AKT signaling pathway. Impaired ERBB2 expression or function has been implicated in the progression of breast and gastric cancers [26], mainly through targeting the PI3K/AKT and MAPK pathways [27]. Previous studies also demonstrated that some DEirlcnRNAs can modulate those pathways such as lncRNA-ZEB2-AS1 and MEF2C-AS1 [24, 28].

In this study, our IRLP signature can reflect the characteristics of the TIME based on the tumor-infiltrating lymphocytes which were adopted to define patients with BRCA as most likely to benefit from ICI therapies. We investigate whether RS was related to the TIME including TIICs, checkpoint-related biomarkers, and immunosuppression-related signal pathways. Research has demonstrated a higher infiltration level of CD8^+^ T cells correlated with a better response to traditional chemotherapy and ICIs in patients with TNBC [29, 30].

By integrating these statistical results, data demonstrated that the low-risk group classified by the IRLP signature was positively associated with a higher infiltration level of CD8^+^ T cells as well as higher expression levels of checkpoint-related biomarkers. Our results indicate that this novel IRLP signature could preciously classify the patients who can benefit from giving any sort of immunotherapy.

Furthermore, the macrophage M2 phenotype follows an immunosuppressive action in general to promote tumor growth. As such, tumors highly infiltrated with macrophage M2 cell [31] and low expression levels of MHC class II related pathway genes were deemed to have a poor prognosis of TNBC [32], where the high-risk group identified by the signature was positively associated with a higher infiltration level of macrophage M2 cell and negatively associated with those MHC-locus embedded genes. The enrichment analysis showed those DEirlncRNAs most related to the T cell receptor signaling pathway which indicated that antigen-presenting progression is essential in the prognosis of BRCA.

The current prediction model had not been implemented in a large patient cohort, and therefore, the predictive power for our proposed gene panel might be overestimated in this molecular-divergent BRCA sample pool. Secondly, the coexpression model relied on carcinoma in situ samples, and this limited the usage of our signature panel aside from conducting a biopsy. In fact, lncRNAs were found to exist in exosomes and also freely circulating form in the bloodstream; therefore, the utility of our panel as a surrogate marker could be expanded if being considered in a liquid biopsy setting.

Finally, we had yet to demonstrate the specificity of our gene signature across different diseases, which might or might not hasten its clinical application as a surrogate biomarker for prognosis in all immune-privileged primary cancer lesions. As much as we would like to develop a specific panel sufficient but limited to stratify BRCA subtypes in the assessment of the suitability of ICIs, there remains a high chance that the same noncoding genes may be useful to all these cancer types towards a unified approach.

## 5. Conclusions

This study successfully constructed an IRLP signature for the predictive prognosis of BRCA patients independent of an individual lncRNA expression. Moreover, our signature reliably predicts individuals who will benefit from the immunotherapy, thus contributing to the development of personalized immunotherapy treatment for BRCA patients.

## Data Availability

The datasets for this study can be found in TCGA (https://tcga-data.nci.nih.gov/tcga/) and ImmPort database (http://www.immport.org).

## Supplementary Materials

Supplementary Materials Tables are uploaded in the supplementary materials files, and details are shown below. Table S1: patient characteristics. A total of 1055 eligible patients with BRCA were recruited for the survival analysis. The characteristics of those patients were shown in this table. Table S2: investigation of tumor-infiltrating immune cells in all subtypes. Since BRCA was identified as 4 subtypes, this table included 4 parts. Part A shows the composition of tumor-infiltrating immune cell profiles in luminal A breast cancer. Part B, part C, and part D show the composition of tumor-infiltrating immune cell profiles in luminal B, basal, and HER2 subtypes, respectively. Table S3: GO enrichment analysis results.
