# Supplementary material for: Machine Learning-Devised Immune-Related lncRNA Signature Panel Predicts the Prognosis and Immune Landscape in Breast Cancer Novel IRLP Signature in BRCA
Source: J Immunol Res. 2022 Aug 18;2022:3704798. doi: 10.1155/2022/3704798 (PMC9410861; doi:10.1155/2022/3704798)
Supplement: Supplementary Materials — Tables are uploaded in the supplementary materials files, and details are shown below. Table S1: patient characteristics. A total of 1055 eligible patients with BRCA were recruited for the survival analysis. The characteristics of those patients were shown in this table. Table S2: investigation of tumor-infiltrating immune cells in all subtypes. Since BRCA was identified as 4 subtypes, this table included 4 parts. Part A shows the composition of tumor-infiltrating immune cell profiles in luminal A breast cancer. Part B, part C, and part D show the composition of tumor-infiltrating immune cell profiles in luminal B, basal, and HER2 subtypes, respectively. Table S3: GO enrichment analysis results. [file 3704798.f1.docx]

Supplementary Table S1. Patient characteristics

| Characteristics | No. of patients | % |
| --- | --- | --- |
| Total number of patients | 1055 |  |
| Age |  |  |
| =<60 | 593 | 56.2 |
| >60 | 462 | 43.8 |
| Median |  |  |
| Gender |  |  |
| Males | 12 | 1.1 |
| Females | 1043 | 98.9 |
| Primary tumor (T) stage |  |  |
| T1 | 270 | 25.6 |
| T2 | 620 | 58.8 |
| T3 | 133 | 12.6 |
| T4 | 32 | 3.0 |
| Regional lymph nodes (N) stage |  |  |
| N0 | 506 |  |
| N1 | 360 |  |
| N2 | 177 |  |
| N3 | 72 |  |
| Metastatic disease |  |  |
| M0 | 888 | 84.2 |
| M1 | 17 | 1.6 |
| Overall stage |  |  |
| Stage I | 179 | 17.0 |
| Stage II | 616 | 58.4 |
| Stage III | 243 | 23.0 |
| Stage IV | 17 | 1.6 |
| Survival status |  |  |
| Alive | 915 | 86.7 |
| Death | 140 | 13.3 |

Supplementary Table S2. Investigation of tumor-infiltrating immune cells by Wilcoxon signed-rank test in all subtypes

1. Luminal A

| Immune cell types | High-risk group | Low-risk group | P-value |
| --- | --- | --- | --- |
| B_memory | 0.000186326 | 0.000851384 | 0.15 |
| B_naive *** | 0.118695869 | 0.150850012 | 0.00038 |
| Dendritic_activated | 0.00235172 | 0.003408106 | 0.43 |
| Dendritic_resting | 0.031152569 | 0.034362098 | 0.63 |
| Eosinophils | 0.002290842 | 0.000308338 | 0.2 |
| Macrophages_M0 | 0.125752152 | 0.119324409 | 0.72 |
| Macrophages_M1 | 0.057932569 | 0.063485706 | 0.23 |
| Macrophages_M2 **** | 0.220663912 | 0.169992442 | 4.8e-05 |
| Mast_activated | 0.000980045 | 0.002761526 | 0.12 |
| Mast_resting | 0.110835119 | 0.099176736 | 0.21 |
| Monocytes * | 0.012239034 | 0.006288346 | 0.043 |
| Neutrophils | 0.002601737 | 0.002191123 | 0.8 |
| NK_activated | 0.024922248 | 0.029521298 | 0.24 |
| NK_resting | 0.00199556 | 0.001485602 | 0.63 |
| Plasma_cells ** | 0.02093954 | 0.036943735 | 0.0025 |
| T_CD4_memory_activated | 0.00417493 | 0.004292791 | 0.94 |
| T_CD4_memory_resting | 0.147106549 | 0.145786418 | 0.89 |
| T_CD4_naive | 0 | 0 | NA |
| T_CD8 | 0.073654073 | 0.083000055 | 0.28 |
| T_follicular_helper | 0.02427077 | 0.024848492 | 0.85 |
| T_gamma_delta | 0.006071062 | 0.007046641 | 0.56 |
| T_regulatory_Tregs | 0.011183113 | 0.014074761 | 0.15 |

1. Luminal B

| Immune cell types | High-risk group | Low-risk group | P-value |
| --- | --- | --- | --- |
| B_memory | 0.001373423 | 0.000685313 | 0.38 |
| B_naive ** | 0.095647952 | 0.110498546 | 0.0056 |
| Dendritic_activated | 0.00361197 | 0.004460354 | 0.53 |
| Dendritic_resting | 0.019483207 | 0.021915526 | 0.49 |
| Eosinophils | 0.000551373 | 0.000196429 | 0.17 |
| Macrophages_M0 | 0.169872582 | 0.155837839 | 0.25 |
| Macrophages_M1 * | 0.064102928 | 0.071028345 | 0.035 |
| Macrophages_M2 **** | 0.251730896 | 0.204510357 | 3e-07 |
| Mast_activated | 0.003069552 | 0.001719849 | 0.25 |
| Mast_resting | 0.08895861 | 0.07775714 | 0.053 |
| Monocytes ** | 0.009150604 | 0.004412958 | 0.0033 |
| Neutrophils * | 0.003625435 | 0.002366168 | 0.03 |
| NK_activated *** | 0.021667073 | 0.029587314 | 0.00097 |
| NK_resting | 0.002898225 | 0.001987373 | 0.33 |
| Plasma_cells ** | 0.017851422 | 0.026793004 | 0.0053 |
| T_CD4_memory_activated ** | 0.007225653 | 0.01300667 | 0.0057 |
| T_CD4_memory_resting | 0.129858977 | 0.128115196 | 0.78 |
| T_CD4_naive **** | 0 | 0 | NA |
| T_CD8 | 0.060096325 | 0.081736162 | 7.9e-05 |
| T_follicular_helper ** | 0.027427848 | 0.031182392 | 0.089 |
| T_gamma_delta **** | 0.008375052 | 0.012755332 | 0.0089 |
| T_regulatory_Tregs | 0.013421052 | 0.019447868 | 5.9e-05 |

1. Basal

| Immune cell types | High-risk group | Low-risk group | P-value |
| --- | --- | --- | --- |
| B_memory | 0.00384155 | 0.00279722 | 0.56 |
| B_naive | 0.096778492 | 0.095018323 | 0.86 |
| Dendritic_activated | 0.01833754 | 0.012252243 | 0.24 |
| Dendritic_resting | 0.015511402 | 0.015523254 | 1 |
| Eosinophils | 0.001707423 | 0.001000832 | 0.42 |
| Macrophages_M0 | 0.229471083 | 0.238658355 | 0.69 |
| Macrophages_M1 | 0.084902725 | 0.095214117 | 0.23 |
| Macrophages_M2 *** | 0.172684434 | 0.130253956 | 0.00063 |
| Mast_activated | 0.007912625 | 0.009648228 | 0.57 |
| Mast_resting | 0.020449324 | 0.016507724 | 0.3 |
| Monocytes ** | 0.008691742 | 0.001693215 | 0.0041 |
| Neutrophils | 0.002525423 | 0.001621651 | 0.29 |
| NK_activated | 0.041582345 | 0.049129348 | 0.16 |
| NK_resting | 0.001947249 | 0.002737542 | 0.58 |
| Plasma_cells | 0.028194861 | 0.030126129 | 0.74 |
| T_CD4_memory_activated | 0.019939411 | 0.032849565 | 0.014 |
| T_CD4_memory_resting | 0.10019377 | 0.083397113 | 0.091 |
| T_CD4_naive | 6.86E-05 | 0 | 0.32 |
| T_CD8 ** | 0.068326361 | 0.096890992 | 0.0037 |
| T_follicular_helper | 0.050009411 | 0.05372956 | 0.45 |
| T_gamma_delta | 0.008784867 | 0.010157864 | 0.57 |
| T_regulatory_Tregs | 0.018138921 | 0.020789343 | 0.39 |

1. HER2

| Immune cell types | High-risk group | Low-risk group | P-value |
| --- | --- | --- | --- |
| B_memory | 0.000994574 | 0.002033544 | 0.49 |
| B_naive | 0.096132198 | 0.110347386 | 0.23 |
| Dendritic_activated | 0.003210767 | 0.004374274 | 0.58 |
| Dendritic_resting | 0.011591294 | 0.013148082 | 0.74 |
| Eosinophils | 0.000149076 | 4.00E-05 | 0.33 |
| Macrophages_M0 | 0.216540098 | 0.186717683 | 0.25 |
| Macrophages_M1 | 0.071277805 | 0.078643071 | 0.29 |
| Macrophages_M2 | 0.207900263 | 0.174590245 | 0.07 |
| Mast_activated | 0.000780887 | 3.02E-05 | 0.34 |
| Mast_resting | 0.06505282 | 0.056744613 | 0.37 |
| Monocytes | 0.002642487 | 0.001291338 | 0.21 |
| Neutrophils | 0.001285095 | 0.001317064 | 0.97 |
| NK_activated | 0.025033698 | 0.035419664 | 0.14 |
| NK_resting | 0.004592346 | 0.000736478 | 0.094 |
| Plasma_cells | 0.026804079 | 0.045335523 | 0.058 |
| T_CD4_memory_activated | 0.015251409 | 0.021139486 | 0.2 |
| T_CD4_memory_resting | 0.119050639 | 0.111441298 | 0.57 |
| T_CD4_naive | 0 | 0 | NA |
| T_CD8 | 0.067206897 | 0.087086913 | 0.11 |
| T_follicular_helper | 0.033807465 | 0.036158472 | 0.65 |
| T_gamma_delta | 0.011664131 | 0.013976039 | 0.5 |
| T_regulatory_Tregs | 0.019032033 | 0.019428119 | 0.91 |

Supplementary Table S3. GO enrichment analysis results

| ID | P-value | Count |
| --- | --- | --- |
| GOBP ANTIGEN RECEPTOR MEDIATED SIGNALING PATHWAY | 2.86E-37 | 25 |
| SMID BREAST CANCER NORMAL LIKE UP | 1.50E-32 | 25 |
| GOBP T CELL RECEPTOR SIGNALING PATHWAY | 1.51E-27 | 18 |
| KEGG T CELL RECEPTOR SIGNALING PATHWAY | 5.91E-24 | 14 |
| GOBP B CELL ACTIVATION | 3.13E-22 | 17 |
| KEGG B CELL RECEPTOR SIGNALING PATHWAY | 8.34E-22 | 12 |
| GOBP B CELL RECEPTOR SIGNALING PATHWAY | 6.80E-21 | 13 |
| GOBP MONONUCLEAR CELL DIFFERENTIATION | 9.21E-19 | 16 |
